# Low-Frequency Ultrasound Effects on Cellulose Nanocrystals for Potential Application in Stabilizing Pickering Emulsions

**DOI:** 10.3390/polym15224371

**Published:** 2023-11-10

**Authors:** Louise Perrin, Stephane Desobry, Guillaume Gillet, Sylvie Desobry-Banon

**Affiliations:** 1 Laboratory of Biomolecules Engineering (LIBio), University of Lorraine, 2 Avenue de la Foret de Haye, BP 20163, 54500 Vandœuvre-les-Nancy, France; stephane.desobry@univ-lorraine.fr (S.D.); sylvie.desobry@univ-lorraine.fr (S.D.-B.); 2 SAS GENIALIS Route d’Acheres, 18250 Henrichemont, France; g.gillet@genialis.fr

**Keywords:** sustainable polymer, liquid crystals, properties, structure, rheological analysis

## Abstract

Cellulose, in the form of cellulose nanocrystals (CNCs), is a promising biomaterial for stabilizing Pickering emulsions (PEs). PEs are commonly formed using low-frequency ultrasound (LFU) treatment and impact CNC properties. The present study investigated the specific effects of LFU treatment on CNCs’ chemical and physical properties. CNCs were characterized using dynamic light scattering, ζ-potential determination, Fourier transform infrared spectroscopy, X-ray diffraction, and contact angle measurement. CNC suspensions were studied using rheological analysis and static multiple light scattering. LFU treatment broke CNC aggregates and modified the rheological behavior of CNC suspensions but did not affect the CNCs’ chemical or crystallographic structures, surface charge, or hydrophilic properties. During the storage of CNC suspensions and PEs, liquid crystal formation was observed with cross-polarized light. Hypotheses related to the impact of liquid crystal CNCs on PE stability were proposed.

## 1. Introduction

Cellulose is a renewable, biodegradable, and biocompatible resource attracting growing interest in many fields. This non-toxic material is the most abundant biopolymer on earth, with an estimated production of between 10^11^ and 10^12^ tons each year. Cellulose is a linear homopolymer composed of several thousands of 1,4-anhydro-D-glucopyranose units (varying depending on cellulose sources). Cellulose chain assemblies, through hydrogen bonds, form nanofibrils and microfibrils [1]. Cellulose can be extracted from wood, plants, algae, marine animals, and fungi, as well as bacteria [2,3].

In plants and wood, cellulose microfibrils form a matrix with hemicelluloses and lignin. Cellulose extraction requires chemical treatments, such as bleaching with sodium chlorite (NaClO_2_) or hydrogen peroxide (H_2_O_2_) to remove lignin and alkaline treatment with sodium hydroxide (NaOH) to remove hemicelluloses [4]. The resulting cellulose microfibrils can then be used to produce nanocelluloses, notably in the form of cellulose nanocrystals (CNCs).

CNCs are mainly extracted with acid hydrolysis using hydrochloric, sulfuric, phosphoric, or organic acids and also with enzymatic hydrolysis, ionic liquid treatment, and subcritical water hydrolysis. These methods can be used in combination with mechanical treatments such as ball milling, ultrasonication, high-pressure homogenization, or microfluidization [1]. CNCs are characterized by their high crystallinity and nanometric size. The diameter of CNCs varies from two to seventy nm, and the length can vary from one hundred to several hundreds of nanometers [3,5].

CNCs are easily produced at the industrial scale [6] with frequent use in many fields such as food, paper, textile, paint, packaging, cosmetic, biomedical, wastewater treatment, energy, and electronic applications [4,5,7,8]. Among their usages, CNCs can stabilize Pickering emulsions (PEs) in cosmetic, food, and pharmaceutical products [9]. PEs are emulsions stabilized by solid particles [10], forming a mechanical barrier at the oil/water interface and preventing droplet coalescence. In addition, particles can form a three-dimensional network between droplets, preventing their displacement and leading to a highly stable emulsion [11]. Although the first works on PEs date back more than a century [12,13], interest in these emulsions has only developed since the early 2000s, and the annual research has increased each year [14,15,16].

The use of inorganic and synthetic particles to stabilize PEs is the most well-known approach, but current challenges are associated with the use of renewable, sustainable, and green colloidal particles. Many particles based on proteins, lipids, and polysaccharides can be used to stabilize PEs [14,17]; among them, CNCs appear to be a promising biomaterial [18]. CNCs are reported to have amphiphilic properties due to the crystalline organization of cellulose, which is responsible for their position at the oil/water interface [19]. PEs formulated with CNCs are stable for several months [20,21].

PEs are most frequently formed using low-frequency ultrasound (LFU) emulsification [22]. Cavitation forces during LFU treatment can impact CNC properties by reducing CNCs’ size and modifying their surface charge or wettability [23,24,25,26,27,28], and these properties determine PE stabilization [29]. For example, a reduced CNC size and increased contact angle (i.e., hydrophobicity) during LFU treatment improved O/W PE stability [27,30]. However, these studies were carried out on non-commercial cellulose and are difficult to replicate, as CNC properties differ in terms of cellulose sources and extraction methods [18].

In a previous paper, we studied PE formation using commercial CNCs [31]. A question arose regarding the specific influence of the LFU process on CNC properties. The aim of the present work was to investigate how chemical and physical CNC properties, modified with LFU treatment, can influence their ability to formulate and stabilize PEs.

## 2. Materials and Methods

### 2.1. Materials

CNCs, produced from wood using sulfuric acid hydrolysis, were purchased from Celluforce (Montreal, Canada). The water used was deionized using an Aquadem purification system (Veolia, Aubervilliers, France) to reach a resistivity of 18 MΩ·cm. Caprylic/capric triglycerides (Miglyol^®^ 812N) were provided by IOI Oleochemical GmbH (Witten, Germany).

### 2.2. CNC Suspension Preparation and Low-Frequency Ultrasound Treatment

CNC suspensions at 0.5 and 5% (*w*/*w*) were formulated by stirring an appropriate CNC quantity in 200 g of water for 12 h at 20°C. The LFU conditions for treating the CNC suspensions were the same as those previously used for PE formation [31]: 125 g of CNC suspension was treated using LFU (20 kHz) with an amplitude of 40% and cycles of 25 s ON and 5 s OFF, using a Fisherbrand™ sonicator 500 W (Fisher Scientific, Waltham, MA, USA). The treatment time was 45 min, which corresponded to a total energy of about 58 kJ. Energy was reported in kJ per gram of CNCs. The LFU treatment was carried out in a thermoregulated reactor to avoid an excessive temperature rise in the suspension (23 ± 1 °C). The CNC suspensions treated using LFU were named “CNCs-US”, with “US” for “ultrasounds”. The remaining untreated CNC suspensions were referred to as “CNCs-UT”, with “UT” for “untreated”.

### 2.3. Size and ζ-Potential Measurements

Size and ζ-potential of CNCs in suspension were measured using dynamic light scattering (DLS) at an angle of 173° with a Zetasizer Nano ZS (Malvern Instruments, Malvern, UK) equipped with a green laser at 532 nm. The DLS method considers particles as spheres, whereas CNCs have a rod-like shape. Thus, calculated data are given as apparent hydrodynamic size to compare CNC samples since measurements were carried out under the same conditions [32,33]. D10, D50, and D90 are size distribution parameters where 10%, 50%, and 90% particles have a smaller apparent diameter, respectively. Analyses were performed in triplicate at 25 °C.

### 2.4. Chemical Structure Analysis

The CNC chemical structures were analyzed using Fourier transform infrared spectroscopy in attenuated total reflection mode (ATR-FTIR) using a TENSOR 27 spectrometer equipped with a deuterated triglycine sulfate (DTGS) detector (Bruker, Germany) and an ATR top-plate (ATR Platinum, Bruker, Germany). The CNC suspensions were previously freeze-dried (Christ Beta 1-8 LD Plus, Martin Christ, Osterode am Harz, Germany) for 72 h at −30 °C and 0.37 mbar. The measurements were performed with 64 scans, 10 kHz scanning, from 4000 to 400 cm^−1^, and with a resolution of 4 cm^−1^. The raw data were processed using OPUS software, version 7.2 (Bruker, Germany). The air spectrum, measured as a reference, was subtracted. H_2_0 and CO_2_ peaks were compensated, and absorbance caused by the diamond was removed by generating a straight line from 2250 to 2440 cm^−1^. The ATR spectra were then converted to absorbance spectra, vector normalization was applied, and a baseline correction was performed. Three measurements for each sample were performed and averaged.

### 2.5. X-ray Diffraction Measurements

X-ray diffraction (XRD) patterns of CNC samples were determined with a D8 ADVANCE X-ray diffractometer (Bruker, Germany) using Cu Kα_1_ radiation (λ = 1.54056 Å). The scattered radiation was detected in the range of 2θ range 5° to 100°. Diffractograms obtained were analyzed with DIFFRAC.EVA software, version 5.2.0.5 (Bruker, Karlsruhe, Germany).

### 2.6. Contact Angle Measurements

Static sessile drop contact angle measurements were carried out to determine CNC hydrophilicity. CNC films were formed using the casting method with 2.5% (*w*/*w*) CNC suspensions. The films were dried in atmosphere at 20 °C and 40% relative humidity for 4 days and then conditioned in a desiccator containing silica gel stored at 20 °C for at least 2 days prior to measurements. The contact angle measurements were performed using an OCA 15EC goniometer (DataPhysics, Filderstadt, Germany) and automatically calculated using the Young–Laplace method. The water contact angle was determined by depositing 4 µL of deionized water on the CNC film surface and measured 10 s after deposition. The results were reported as the average of five measurements.

### 2.7. Rheological Measurements

An HR20 discovery hybrid rheometer (TA Instruments, New Castle, DE, USA) using a 50 mm parallel plate geometry and a gap height of 250 µm between the two plates was used to perform rheological measurements. Before starting a measurement, an equilibration time of 180 s was set. Viscosity measurements were carried out over a shear rate range from 0.01 to 1000 s^−1^ in steady-state mode. The storage modulus (G′) and loss modulus (G″) were determined with a dynamic frequency sweep in the angular frequency range from 1 to 100 rad.s^−1^ at a constant strain of 1.0% (previously determined to be in the linear viscoelastic range). All measurements were performed at 20 ± 0.1 °C on fresh CNC suspensions. The rheological results were reported as the mean of at least two measurements.

### 2.8. Observation under Cross-Polarized Light

The presence of isotropic and anisotropic phases in CNC suspensions was observed under cross-polarized light, using a method adapted from Browne et al. (2022) [34]. Briefly, 0.5 to 5% (*w*/*w*) CNC suspensions were prepared with successive dilution of the 5% *w*/*w* CNC suspension. Six grams of each CNC suspension were placed in a glass bottle. After 4 weeks of settling at 20 °C, the samples were placed between two crossed linear polarizers to be photographed. The anisotropic phase ratio (%) was determined as the ratio between the height of the anisotropic phase and the total height of the suspension, measured with ImageJ software, version 1.53t (National Institutes of Health, Bethesda, MD, USA).

### 2.9. Suspension Stability Study

The CNC suspension stability was studied using static multiple light scattering measurements with a Turbiscan Classic MA2000 apparatus (Formulaction, Toulouse, France) using a pulsed near-infrared light source at 850 nm. Seven milliliters of CNC suspensions were placed in a specific glass cell, which was then scanned with a light source. Detection was controlled with a transmission detector, which measured light that passed through the sample at 180° to the incident beam. The measurements were carried out in duplicate for up to 56 days of storage at 20 °C.

### 2.10. PE Preparation and Characterization

PEs, containing 1 or 5% of CNCs (w/w in the final emulsion) and 10% oil, were formulated in two steps [31]. The CNCs-UT suspension and oil (caprylic/capric triglycerides) were pre-emulsified using high-speed homogenization for 5 min at 10,000 rpm, with an Ultra-turrax^®^ T-25 equipped with a S 25N-18G dispersal tool (IKA-Werke, Staufen im Breisgau, Germany). Emulsification was then carried out using LFU under the same conditions as for CNC suspension processing (Section 2.2), i.e., a frequency of 20 kHz, an amplitude of 40%, and cycles of 25 s ON and 5 s OFF. The PE temperature was maintained at 23 ± 1 °C during LFU treatment.

The PE structure was observed using an Olympus BX51 microscope (Olympus, Tokyo, Japan) equipped with an AxioCam ICc 1 CCD camera (Zeiss, Oberkochen, Germany).

### 2.11. Statistical Analysis

Data were analyzed with one-way analysis of variance (ANOVA) using RStudio software, version 1.3.959 (R-tools Technology, Richmond Hill, ON, Canada). Differences at *p* < 0.05 were considered significant.

## 3. Results and Discussion

### 3.1. LFU Effect on CNC Size

**Figure 1:**
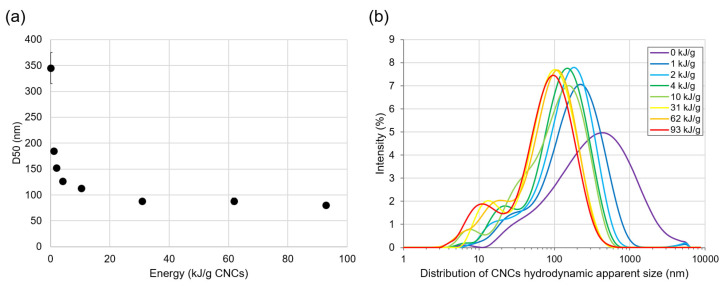
Size study of CNCs suspended at 0.5% (*w*/*w*) and treated using LFU for 45 min. (**a**) Apparent hydrodynamic D50 of CNCs and (**b**) mean distribution of the CNCs’ apparent hydrodynamic diameter as a function of energy applied with LFU treatment. Low standard deviations of apparent hydrodynamic D50 are reported in (**a**).

The LFU treatment effects on CNC size were studied using DLS measurement. Figure 1a shows that the CNCs’ apparent hydrodynamic D50 decreased rapidly from 350 to 80 nm when LFU was applied up to 30 kJ/g CNCs. This could be attributed to the disaggregation of large CNC particles due to LFU-induced cavitation [33]. For LFU treatments up to 93 kJ/g CNCs, a plateau was observed around 80 nm (Figure 1a). However, the CNCs’ apparent hydrodynamic diameter distribution showed the emergence of 10 nm particles with increasing LFU energy (Figure 1b). This demonstrates the capacity of LFU treatment to break up CNCs in particles of 10 nm, as previously observed [33].

The results reported in Figure 1 concern LFU treatment for the 0.5% (*w*/*w*) CNC suspension. Similar results were obtained for the 5% (*w*/*w*) CNC suspension. Size distribution parameters are presented in Table 1. Even if the energy per gram of CNCs was 10 times lower for the 5% (*w*/*w*) CNC suspension, D50 and D90 were slightly increased, while D10 remained unchanged. These results showed that very fine particle formation and CNC breakup were due to the energy increase. The agglomerated CNC breakage was more governed by the ultrasound power than by the energy supplied per gram of CNCs. These observations were in agreement with those obtained by Beuguel et al. (2018) [23].

### 3.2. LFU Effect on the Chemical and Crystallographic Properties of CNCs

**Figure 2:**
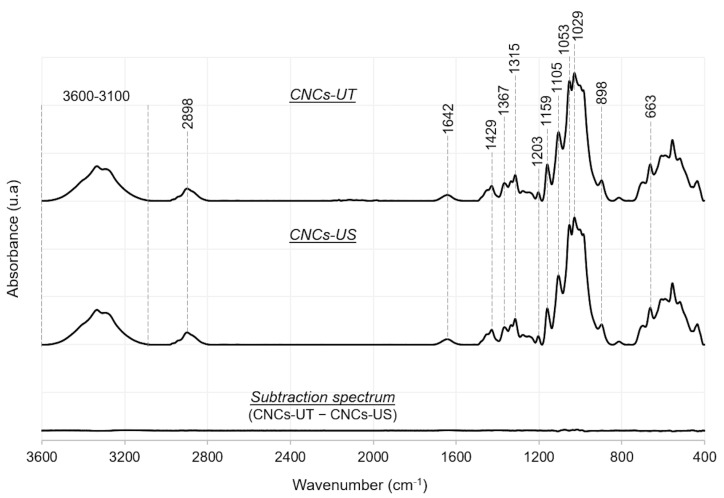
FTIR spectra of CNCs-UT and CNCs-US, and subtraction spectrum (CNCs-UT − CNCs-US).

An FTIR analysis was carried out to investigate the CNCs’ chemical composition and possible chemical changes due to LFU treatment (Figure 2). From all the identified peaks (Table 2), those observed at 1029 cm^−1^ (C-O-C deformation) and 1367 cm^−1^ (C-H asymmetric stretching and C-O symmetric stretching) were characteristic of pyranose rings of glucose units [35,36]. Glycosidic linkages between glucose units were highlighted by peaks at 1159 and 1105 cm^−1^ corresponding to C-O-C wagging and twisting and C-O-C asymmetric stretching, respectively [32,35]. The peak at 898 cm^−1^ was assigned to C_1_-H rocking and O-H bending [36]. The peak at 1203 cm^−1^ (S=O stretching) referred to the presence of sulfate groups [36], which originate from acid hydrolysis of cellulose in CNCs using sulfuric acid [37].

The FTIR spectra were similar for CNCs-UT and CNCs-US, showing that no change in the CNCs’ chemical structure was caused by LFU treatment, particularly with regard to the presence of sulfate groups. Even if CNC suspensions were thermo-regulated during LFU treatment, hot-local points might rise and cause the de-esterification of sulfate groups [41]. The FTIR results ensured that LFU treatment had no significant effect on the CNCs’ chemical structure. In addition, the absence of a peak shift also showed that LFU treatment did not change the cellulose polymorphism [42].

**Figure 3:**
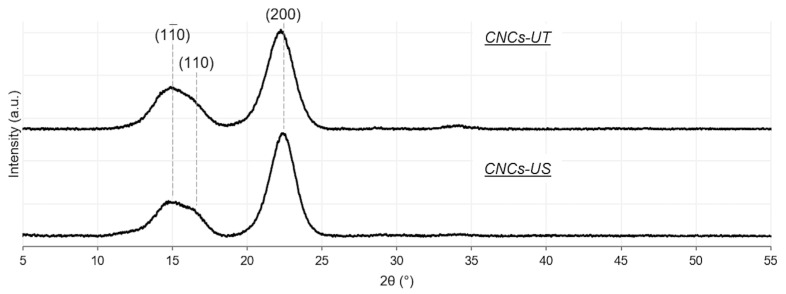
X-ray diffraction patterns of CNCs-UT and CNCs-US.

XRD measurements were carried out to investigate the treatment effect of LFU on the CNCs’ structure. The CNCs-UT diffractogram revealed three peaks around 2θ = 15°, 16.5° and 22.8° corresponding to (1$\bar{1}$0), (110) and (200) crystallographic planes, respectively (Figure 3). These planes were characteristic of cellulose I [43]. The CNCs-US diffractogram was similar to that of CNCs-UT, showing that the CNCs’ structure was not altered with LFU treatment, in agreement with the results of Zakani et al. (2022) [33]. The (200) crystallographic plane would be composed solely of CH groups, conferring hydrophobic properties, while the other (1$\bar{1}$0) and (110) planes would be hydrophilic [19]. The XRD results not only confirmed the FTIR results but also made it possible to identify the different crystallographic planes of the CNCs.

### 3.3. LFU Effect on CNC Charge

The LFU effect on CNC charge was studied using ζ-potential measurements. CNCs-UT and CNCs-US exhibited a negative ζ-potential of −50.25 ± 1.33 mV and −53.20 ± 3.22 mV, respectively, due to the presence of sulfate groups, as identified with the FTIR measurements. ζ-potential values above |30 mV| suggested that CNC colloidal suspensions were stable over time, as charges are sufficient for CNC particles to repel each other [4,32]. Although Chen et al. (2014) and Csiszar et al. (2016) observed a slight increase in ζ-potential value with increasing sonication time [44,45], no significant change in ζ-potential was observed between both CNC samples, in accordance with Beuguel et al. (2018) [23]. This suggested that the LFU treatment did not break covalent ester bonds between the sulfate groups and cellulose, supporting the FTIR results (Section 3.2.). Moreover, the pH of the CNC suspensions (close to 6.3 for 0.5% (*w*/*w*) CNC suspensions) was not significantly altered with LFU treatment, confirming that no sulfuric acid molecule was released into the medium.

### 3.4. LFU Effect on CNC Hydrophilicity

Particle wettability is a key factor in PE stability and is characterized by contact angle measurements [46]. The contact angles for CNCs-UT (θ_CNCs-UT_) and CNCs-US (θ_CNCs-US_) were determined using the sessile drop method on CNC films. It can be noticed that this method did not consider the CNCs’ position at the oil/water interface since in PEs, free CNCs could orient themselves at the interface. However, the sessile drop method provided information on a possible CNC surface hydrophilicity modification with LFU.

Contact angle measurements around 30° revealed the hydrophilic nature of CNCs. No significant difference between CNCs-UT (θ_CNCs-UT_ = 31.9 ± 2.1°) and CNCs-US (θ_CNCs-US_ = 29.1 ± 4.2°) demonstrated that the LFU treatment did not modify the CNCs’ wettability. According to the FTIR and XRD results, neither sulfate groups nor crystalline organization were modified with the LFU treatment. Thus, as expected, hydrophilicity was found to be similar for CNCs-UT and CNCs-US. Our results differed from those obtained by Ni et al. (2021, 2022) who observed an increase in contact angle (hydrophobicity) with LFU treatment time, probably due to CNC fragmentation exposing more hydrophobic crystal planes [27,30]. The CNCs used in this study differed in terms of origin and production methods from those used by Ni et al. (2021, 2022). Thus, the absence of modification in the CNC contact angle confirmed that the LFU treatment did not break CNCs but released aggregated CNC units in our suspensions.

### 3.5. LFU Effect on Rheological Properties

**Figure 4:**
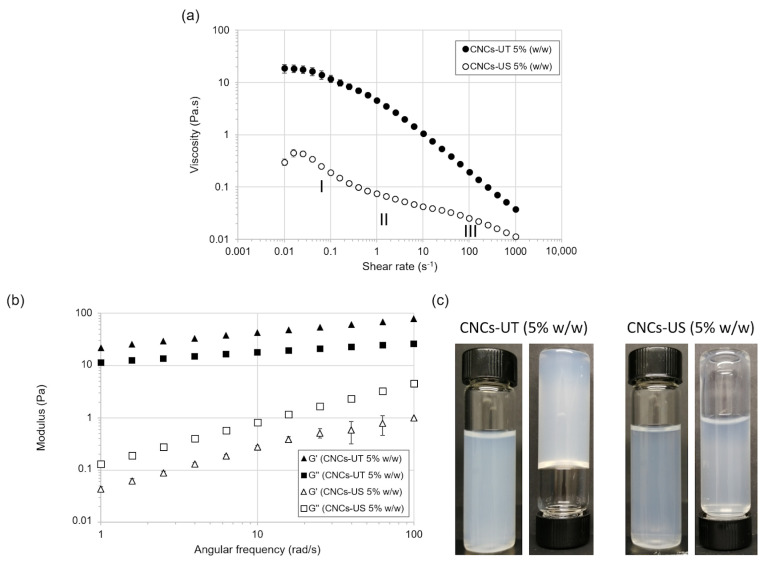
Effect of LFU on the (**a**) viscosity and (**b**) viscoelastic properties of the 5% (*w*/*w*) CNC suspension. (**c**) Visual observations of the CNCs-UT and CNCs-US suspensions.

The rheological properties of 5% (*w*/*w*) CNCs-UT and CNCs-US suspensions were analyzed by measuring viscosity and viscoelastic parameters. The viscosity of the CNC suspensions decreased with increasing shear rate, showing that CNC suspensions had a shear-thinning behavior (Figure 4a). The CNCs-US suspension had a lower viscosity than the CNCs-UT suspension. These results were previously observed and explained in terms of water and ion release during the disruption of CNC aggregates with LFU treatment. Firstly, the entrapment of water molecules in CNC agglomerates would lead to an increase in the CNCs’ apparent concentration, and hence to an increase in viscosity [23,47]. Secondly, the breakage of CNC agglomerates would release ions in the bulk and consequently compress the CNCs’ electrical double layer, reducing electroviscous effects and decreasing the viscosity [23,48].

The viscosity curve of the CNCs-US suspension showed three different phases representative of CNC aqueous suspensions and characteristic of a lyotropic liquid crystalline polymer [49]. At low shear rates, the viscosity decreased due to the progressive alignment of liquid crystal (LC) domains (Phase I). The alignment of all LC domains in the shear direction may correspond to the viscosity stabilization at an intermediate shear rate (Phase II). At higher shear rates, the LCs’ destruction and individual CNC particles’ alignment explained the decrease in viscosity (Phase III). Although the LCs’ presence in 5% (*w*/*w*) CNCs-US suspension was suggested, the rheological profile did not explain if this suspension contained only a liquid crystalline phase or a biphasic phase (i.e., a liquid crystalline phase and an isotropic phase) [50].

LFU treatment also had a strong effect on viscoelastic properties (Figure 4b). For CNCs-UT at 5% (*w*/*w*), the storage modulus (G′) was higher than the loss modulus (G′′), over the whole angular frequency range studied, which is characteristic of a viscoelastic solid. CNCs-US at 5% (*w*/*w*) behaved like a viscoelastic liquid, with G′ lower than G′′ (Figure 4c).

The rheological data showed that LFU favored the dispersion of CNC aggregates and prevented gelation. LFU provided favorable conditions for LC formation. This change in rheological behavior caused by the LFU treatment had already been observed for highly concentrated suspensions [24]. For lower CNC concentrations, suspensions that were not treated with LFU had a viscoelastic liquid behavior [49].

### 3.6. LFU Effect on Liquid Crystal Formation

**Figure 5:**
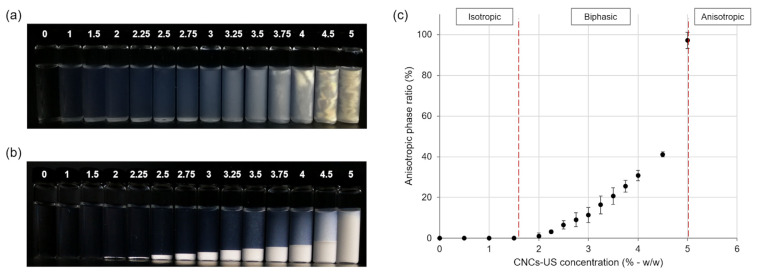
Observation of LCs in CNC suspensions under cross-polarized light. Photographs of (**a**) the CNCs-UT and (**b**) CNCs-US suspensions at different concentrations (in % *w*/*w*) and after 28 days of storage. (**c**) Phase diagram showing evolution in the anisotropic phase ratio versus CNCs-US concentration.

The LCs’ presence in CNCs-US was suggested by the rheological measurements (Figure 4a) in the 5% (*w*/*w*) CNC suspension. Indeed, CNCs have the property of forming chiral nematic LCs above a critical concentration [51]. LC formation was then analyzed over a wide range of CNC concentrations using observations under cross-polarized light. Anisotropic LCs possess specific optical properties that enable them to change the light polarization [52]. As CNC suspensions were observed under cross-polarized light, the LCs were bright, and the isotropic phase appeared as dark [53]. Figure 5a,b highlights LC formation in both CNCs-US and CNCs-UT suspensions. The rheological analysis did not reveal the presence of LCs in CNCs-UT, probably due to the gel-like structure of these suspensions, or possibly due to a lower LC content in the CNCs-UT suspension. Indeed, CNCs-UT formed aggregates (Figure 1) that could disrupt the LCs’ organization.

LCs have the particularity of having a higher density than isotropic phase [54]. After 28 days of storage, they sedimented in the CNCs-US suspensions. In the CNCs-UT suspensions, LCs were dispersed in the whole suspension, due to the gel-like behavior of highly concentrated suspensions, preventing sedimentation. LC formation increased with CNC concentration. The LCs’ sedimentation for CNCs-US enabled macroscopic measurement of the anisotropic phase ratio for each CNC concentration and phase transition diagram build-up (Figure 5c). The isotropic phase was observed up to a critical CNC concentration between 1.5 and 2% (*w*/*w*), where the biphasic suspension was formed with isotropic and anisotropic phases. The 5% (*w*/*w*) CNCs-US suspension was mostly anisotropic.

### 3.7. LFU Effect on CNC Suspension Stability

**Figure 6:**
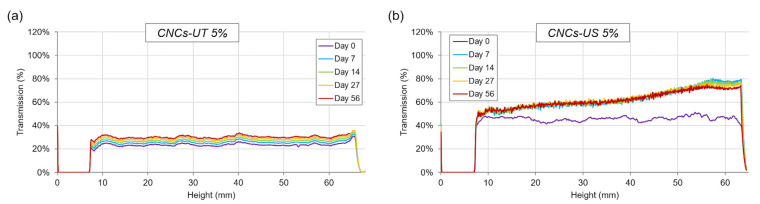
Static multiple light scattering analysis showing transmission data versus sample height (7 to 64 mm) and storage time for the (**a**) CNCs-UT and (**b**) CNCs-US suspensions at 5% (*w*/*w*).

The static multiple light scattering method was used to compare the stability of the CNCs-UT and CNCs-US suspensions at 5% (*w*/*w*) during storage (Figure 6). When the CNC suspension at 5% (*w*/*w*) was treated using LFU, the transmitted light intensity doubled compared with CNCs-UT. This is in accordance with the CNC particle size reduction observed after LFU treatment (Table 1) [47] and consistent with results obtained by Csiszar et al. (2016), who observed that a 3.4% (*w*/*w*) CNC suspension was less opaque after LFU treatment [45]. 

Uniform transmittance from the bottom to the top of the CNCs-UT suspension (Figure 6a) showed that the suspension was stable in relation to its gel structure (Figure 4). The gel aging explained the slight up-translation in transmittance during storage. In contrast with CNCs-UT, the transmitted light intensity of CNCs-US increased significantly from the bottom to the top of the tube at 7 days of storage and would reflect the LC sedimentation and LC concentration gradient over the entire height of the suspension. Multiple light scattering analyses provided additional information on LC gradient within suspension that was not observable under polarized light.

### 3.8. LFU Effect on CNCs and Impact on PE Properties

After studying the LFU effect on CNC properties, the link between CNC properties and PE formation and stabilization, was studied to evaluate how LFU-induced CNC modifications impact PE properties.

#### 3.8.1. Effect of CNC Particle Size and Shape on PEs

CNC particle size plays a role in PE formation and stability. The small particles adsorbed faster at the oil/water interface and formed a denser layer at the interface than larger particles [55]. By reducing the CNCs’ size (Figure 1), the LFU treatment favored CNC adsorption and organization at the oil/water interface and PE formation.

If particles are considered as spheres, particle desorption energy (${\Delta E}_{\mathrm{Desorption}}$) corresponds to Equation (1) [56]:

(1) $${\Delta E}_{\mathrm{Desorption}}{=\pi \;R}^{2}{\;\gamma }_{\mathrm{ow}}\;{\left(1+\cos\theta \right)}^{2},$$

where R is the particle radius, $\gamma_{\mathrm{ow}}$ is the oil–water interfacial tension, and θ is the contact angle of particles measured in the aqueous phase. Although decreasing particle size would favor desorption, Berton-Carabin and Schroën, (2015) highlighted that PEs remained stable because ${\Delta E}_{\mathrm{Desorption}}$ remained high with a value superior to thermal energy at 25 °C [14]. So, decreasing the sphere-like particle size would have little impact on PE destabilization.

Desorption energy also depends on particle shape. For non-spherical particles, length and width have to be considered [57]. The commercial CNCs used in this study have a rod-like shape [34,58]. Rod-like particles are expected to present more physical interactions at interfaces than volume-equivalent spherical particles. LFU, favoring the dispersion of CNC units with rod-like shapes, would act positively on PE stabilization.

#### 3.8.2. Effect of the CNC Particles’ Amphiphilic Properties and Charge on PEs

The XRD results showed that CNCs were composed of hydrophobic and hydrophilic planes (Figure 3) and explained the amphiphilic properties of CNCs and their potential use for stabilizing PEs [19]. LFU treatment would not affect the CNCs’ amphiphilic properties and would not modify the CNCs’ abilities to form a layer at the oil/water interface to stabilize PEs. Although CNCs had amphiphilic properties, contact angle measurements (around 30°) revealed that CNCs are hydrophilic and better stabilize O/W PEs.

Particle surface charge is a key parameter in PE stability and promotes electrostatic repulsion between droplets, preventing droplet coalescence. A high particle surface charge can induce electrostatic repulsion between particles and alter their absorption at the oil/water interface. On the contrary, a low surface charge favors particle aggregation and droplet attraction, leading to coalescence and PEs’ destabilization [59]. According to the literature, the range of ζ-potential values efficient on PE stability is large and depends on CNC origin and PE formulation and processing. PEs with 20% (*w*/*w*) corn oil and 0.6% (*w*/*w*) CNCs (from cotton) were produced using high-speed homogenization for CNC ζ-potential adjusted to −38 mV and −24 mV with NaCl addition [60]. CNCs, extracted from palm-pressed fiber with ζ-potential values around −60 mV, were able to stabilize up to 14-day high-pressure homogenized PEs composed of 20% (*w*/*w*) palm kernel olein and 0.15 or 0.30% CNCs (*w*/*w*) [61]. With a ζ-potential around −50 mV, Celluforce CNCs (as used in this study) stabilized PEs with salt addition [62,63,64,65,66] or without salt [31,67]. As an example, PEs composed solely of water, caprylic/capric triglycerides, and CNCs were shown to be stable for up to 56 days of storage without creaming when the CNC content was above 3% (*w*/*w*) and the oil mass fraction was below 50% (*w*/*w*). PEs with reversible creaming were obtained when the CNC content and the oil mass fraction were less than 3% (*w*/*w*) and 40% (*w*/*w*), respectively [31].

#### 3.8.3. LC Effect on PE Stability

**Figure 7:**
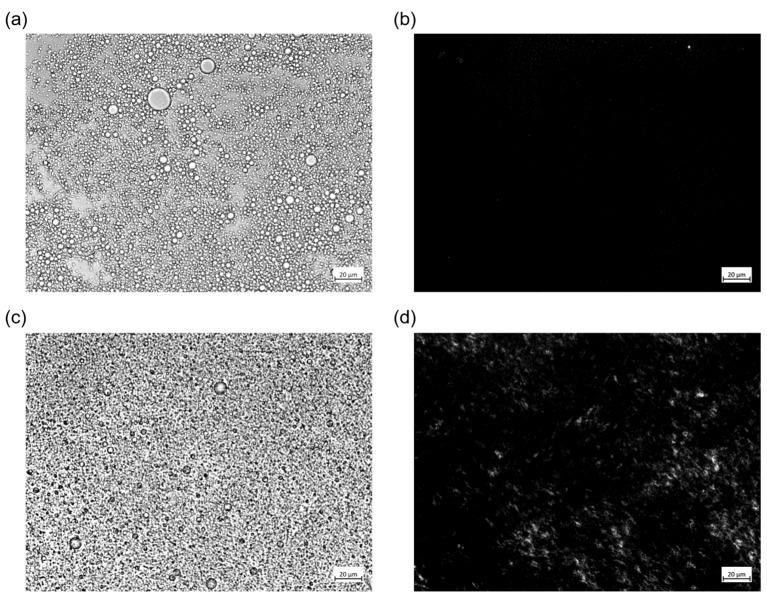
Optical microscope images of PEs containing (**a**,**b**) 1% CNCs (*w*/*w*) and 10% (*w*/*w*) oil and (**c**,**d**) 5% CNCs (*w*/*w*) and 10% (*w*/*w*) oil under (**a**,**c**) unanalyzed polarized light and (**b**,**d**) analyzed polarized light. The scale bar corresponds to 20 µm.

The LCs’ presence in the CNC suspensions influenced the PEs’ formation and stabilization. After LFU processing, the PEs’ structure was observed using a polarized optical microscope (Figure 7). As LCs did not form below 2% CNCs (*w*/*w*) (Figure 5), the PE containing 1% (*w*/*w*) CNCs (Figure 7a) did not reveal any LC bright structures (Figure 7b). On the contrary, in the PE containing 5% (*w*/*w*) CNCs (Figure 7c), bright stains were observed, highlighting the LCs’ presence (Figure 7d). Bright LCs appeared dispersed in a continuous phase, suggesting that CNC concentration was greater than 2% (*w*/*w*). Moreover, the PE was very stable during 56 days of storage without creaming [31].

LCs have already been observed in PEs containing CNCs [68] and other solid particles [69]. The relationship between CNC structuration in LC form and PE stabilization may be relevant. According to the phase diagram of PEs [31] and the present results, a phenomenological model can be proposed, enhancing the LCs’ positive influence on PE formation and stabilization.

**Figure 8:**
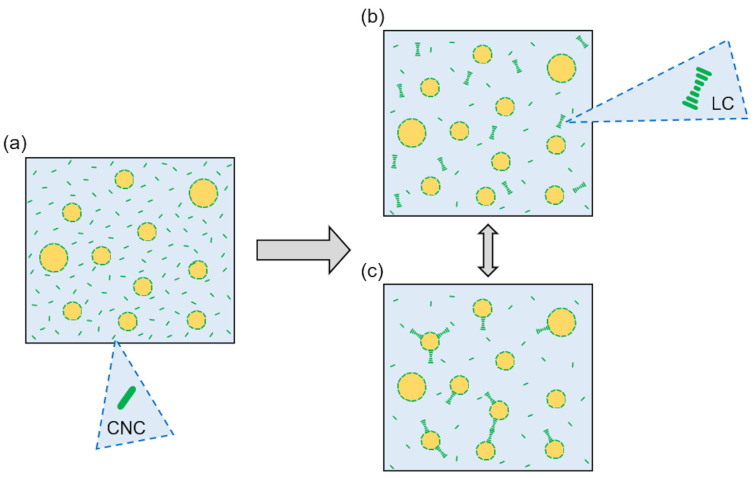
Phenomenological model on the organization of high CNC concentration in PEs. (**a**) Oil droplets prevent LC formation, and CNCs are dispersed in a continuous phase. (**b**) CNCs form LCs dispersed in a continuous phase. (**c**) LCs are dispersed in a continuous phase and adsorb at the oil/water interface.

During LFU processing, CNC units formed a layer at the oil/water interface and stabilized oil droplets [11]. The unadsorbed CNCs (in excess) remained in an aqueous continuous phase (Figure 8a), and their self-organization formed LCs. The LCs were dispersed in the continuous aqueous phase (Figure 8b), and/or adsorbed at the oil/water interface (Figure 8c). The LCs’ formation could favor the droplet stabilization by producing steric repulsion at the droplet interface. It can be supposed that for a high volume fraction (oil and CNCs), a weak LC network could be produced within an aqueous continuous phase.

In our previous study [31], it was shown that PEs containing less than 3% (*w*/*w*) CNCs were unstable with creaming occurring during storage, whereas PEs containing more than 3% (*w*/*w*) remained stable for up to 56 days of storage. Better PE stability with increasing CNC content was assumed to be due to an increasing continuous phase viscosity. The presence of LCs in PEs containing large CNC concentrations acted as an additional stabilizing agent.

## 4. Conclusions

In this study, the LFU treatment effects on CNC properties were investigated with the aim of using CNCs to stabilize PEs. CNC suspensions were treated (or not) using LFU, and their chemical and physical properties were studied and compared.

The CNCs’ size showed that CNC aggregates were dissociated with LFU treatment above 20 kJ/g CNCs. The FTIR results revealed the presence of sulfate groups due to CNC hydrolysis conditions. These sulfate groups provided a high CNC surface charge. In PEs, droplet coalescence was prevented by efficient electrostatic repulsion between them. The XRD results highlighted the presence of a hydrophobic crystallographic plane, conferring amphiphilic properties to CNCs and enabling CNCs to adsorb at the oil/water interface to stabilize PEs. No chemical and crystallographic changes were observed with FTIR and XRD measurements after LFU treatments of CNC suspension. The surface charge and hydrophilic properties of CNCs were not altered with LFU treatment.

However, the LFU treatment modified the rheological properties of CNC suspensions, breaking down gel structures and promoting LC formation and sedimentation. In PEs, when CNCs were in excess in a continuous phase, LCs were formed and participated in PE stabilization. A phenomenological model was proposed to describe LCs’ role in PE stabilization.

The LFU treatment, as a common emulsification process, would not alter CNC properties and could even improve CNCs’ capabilities to adsorb at the oil/water interface and stabilize PEs. Further investigation will be required to validate these hypotheses using LFU-treated CNCs and also CNCs subjected to other emulsification processes like high-pressure homogenization or microfluidization.

## Figures and Tables

**Table 1:** CNC apparent size distribution parameters (D10, D50, and D90) depending on the CNC concentration (0.5 or 5% *w*/*w*) and the energy applied (before or after 45 min LFU treatment).

CNC Suspension Concentration (%—*w*/*w*)	Energy (kJ)	Energy (kJ/g CNCs)	D10 (nm)	D50 (nm)	D90 (nm)
0.5	0	0	59 ± 9.3 ^a^	345 ± 30.0 ^a^	1350 ± 155 ^a^
	58	93	13 ± 1.2 ^b^	80 ± 2.1 ^b^	203 ± 22.7 ^b^
5	0	0	57 ± 2.6 ^a^	366 ± 7.6 ^a^	1370 ± 260 ^a^
	58	9.3	12 ± 1.7 ^b^	100 ± 5.6 ^c^	269 ± 19.8 ^c^

Different superscript letters (^a,b,c^) in the same column indicate significant differences (*p* < 0.05).

**Table 2:** Peak wavenumber and assignment of corresponding chemical groups, identified with FTIR measurements.

Wavenumber (cm^−1^)	Assignments	References
Between 3100 and 3600	O-H stretching of primary and secondary hydroxyl groups	[32,36,38]
2898	C-H stretching	[32,35]
1642	O-H bending of adsorbed water	[32,35,36]
1429	In-plane bending vibration of H-C-H and O-C-H bonds	[38]
1367	C-H asymmetric stretching and C-O symmetric stretching	[35]
1315	C-H_2_ wagging	[39]
1203	S=O stretching	[36]
1159	C-O-C asymmetric stretching	[32,36]
1105	C-O-C wagging and twisting	[35]
1053	C-O stretching	[38]
1029	C-O-C deformation	[36]
898	C-H rocking and O-H bending	[36,40]
663	Out-plane hydrogen bonded O–H groups twisting	[32]

## Data Availability

The data presented in this study are available on request from the corresponding author.

## References

[1] Trache D., Hazwan Hussin M., Mohamad Haafiz M.K., Kumar Thakur V. (2017). Recent Progress in Cellulose Nanocrystals: Sources and Production. Nanoscale.

[2] Nechyporchuk O., Belgacem M.N., Bras J. (2016). Production of Cellulose Nanofibrils: A Review of Recent Advances. Ind. Crops Prod..

[3] Rana A.K., Frollini E., Thakur V.K. (2021). Cellulose Nanocrystals: Pretreatments, Preparation Strategies, and Surface Functionalization. Int. J. Biol. Macromol..

[4] Nagarajan K.J., Ramanujam N.R., Sanjay M.R., Siengchin S., Surya Rajan B., Sathick Basha K., Madhu P., Raghav G.R. (2021). A Comprehensive Review on Cellulose Nanocrystals and Cellulose Nanofibers: Pretreatment, Preparation, and Characterization. Polym. Compos..

[5] Trache D., Tarchoun A.F., Derradji M., Hamidon T.S., Masruchin N., Brosse N., Hussin M.H. (2020). Nanocellulose: From Fundamentals to Advanced Applications. Front. Chem..

[6] Vanderfleet O.M., Cranston E.D. (2021). Production Routes to Tailor the Performance of Cellulose Nanocrystals. Nat. Rev. Mater..

[7] Grishkewich N., Mohammed N., Tang J., Tam K.C. (2017). Recent Advances in the Application of Cellulose Nanocrystals. Curr. Opin. Colloid Interface Sci..

[8] Seddiqi H., Oliaei E., Honarkar H., Jin J., Geonzon L.C., Bacabac R.G., Klein-Nulend J. (2021). Cellulose and Its Derivatives: Towards Biomedical Applications. Cellulose.

[9] Meirelles A.A.D., Costa A.L.R., Cunha R.L. (2020). Cellulose Nanocrystals from Ultrasound Process Stabilizing O/W Pickering Emulsion. Int. J. Biol. Macromol..

[10] Chevalier Y., Bolzinger M.-A. (2013). Emulsions Stabilized with Solid Nanoparticles: Pickering Emulsions. Colloids Surf. A Physicochem. Eng. Asp..

[11] Cui F., Zhao S., Guan X., McClements D.J., Liu X., Liu F., Ngai T. (2021). Polysaccharide-Based Pickering Emulsions: Formation, Stabilization and Applications. Food Hydrocoll..

[12] Pickering S.U. (1907). CXCVI.—Emulsions. J. Chem. Soc. Trans..

[13] Ramsden W., Gotch F. (1904). Separation of Solids in the Surface-Layers of Solutions and ‘Suspensions’ (Observations on Surface-Membranes, Bubbles, Emulsions, and Mechanical Coagulation).—Preliminary Account. Proc. R. Soc. Lond..

[14] Berton-Carabin C.C., Schroën K. (2015). Pickering Emulsions for Food Applications: Background, Trends, and Challenges. Annu. Rev. Food Sci. Technol..

[15] Gonzalez Ortiz D., Pochat-Bohatier C., Cambedouzou J., Bechelany M., Miele P. (2020). Current Trends in Pickering Emulsions: Particle Morphology and Applications. Engineering.

[16] Xiao J., Li Y., Huang Q. (2016). Recent Advances on Food-Grade Particles Stabilized Pickering Emulsions: Fabrication, Characterization and Research Trends. Trends Food Sci. Technol..

[17] Ribeiro E.F., Morell P., Nicoletti V.R., Quiles A., Hernando I. (2021). Protein- and Polysaccharide-Based Particles Used for Pickering Emulsion Stabilisation. Food Hydrocoll..

[18] Dai H., Wu J., Zhang H., Chen Y., Ma L., Huang H., Huang Y., Zhang Y. (2020). Recent Advances on Cellulose Nanocrystals for Pickering Emulsions: Development and Challenge. Trends Food Sci. Technol..

[19] Kalashnikova I., Bizot H., Cathala B., Capron I. (2012). Modulation of Cellulose Nanocrystals Amphiphilic Properties to Stabilize Oil/Water Interface. Biomacromolecules.

[20] Aw Y.Z., Lim H.P., Low L.E., Surjit Singh C.K., Chan E.S., Tey B.T. (2022). Cellulose Nanocrystal (CNC)-Stabilized Pickering Emulsion for Improved Curcumin Storage Stability. LWT.

[21] Ni Y., Li J., Fan L. (2020). Production of Nanocellulose with Different Length from Ginkgo Seed Shells and Applications for Oil in Water Pickering Emulsions. Int. J. Biol. Macromol..

[22] Gauthier G., Capron I. (2021). Pickering Nanoemulsions: An Overview of Manufacturing Processes, Formulations, and Applications. JCIS Open.

[23] Beuguel Q., Tavares J.R., Carreau P.J., Heuzey M.-C. (2018). Ultrasonication of Spray-and Freeze-Dried Cellulose Nanocrystals in Water. J. Colloid Interface Sci..

[24] Gicquel E., Bras J., Rey C., Putaux J.-L., Pignon F., Jean B., Martin C. (2019). Impact of Sonication on the Rheological and Colloidal Properties of Highly Concentrated Cellulose Nanocrystal Suspensions. Cellulose.

[25] Metzger C., Drexel R., Meier F., Briesen H. (2021). Effect of Ultrasonication on the Size Distribution and Stability of Cellulose Nanocrystals in Suspension: An Asymmetrical Flow Field-Flow Fractionation Study. Cellulose.

[26] Xu H.-N., Tang Y.-Y., Ouyang X.-K. (2017). Shear-Induced Breakup of Cellulose Nanocrystal Aggregates. Langmuir.

[27] Ni Y., Li J., Fan L. (2021). Effects of Ultrasonic Conditions on the Interfacial Property and Emulsifying Property of Cellulose Nanoparticles from Ginkgo Seed Shells. Ultrason. Sonochem..

[28] Meirelles A.A.D., Costa A.L.R., Cunha R.L. (2020). The Stabilizing Effect of Cellulose Crystals in O/W Emulsions Obtained by Ultrasound Process. Food Res. Int..

[29] Ming Y., Xia Y., Ma G. (2022). Aggregating Particles on the O/W Interface: Tuning Pickering Emulsion for the Enhanced Drug Delivery Systems. Aggregate.

[30] Ni Y., Wu J., Jiang Y., Li J., Fan L., Huang S. (2022). High-Internal-Phase Pickering Emulsions Stabilized by Ultrasound-Induced Nanocellulose Hydrogels. Food Hydrocoll..

[31] Perrin L., Desobry-Banon S., Gillet G., Desobry S. (2023). Phase Diagram of Pickering Emulsions Stabilized by Cellulose Nanocrystals. Polymers.

[32] Foster E.J., Moon R.J., Agarwal U.P., Bortner M.J., Bras J., Camarero-Espinosa S., Chan K.J., Clift M.J., Cranston E.D., Eichhorn S.J. (2018). Current Characterization Methods for Cellulose Nanomaterials. Chem. Soc. Rev..

[33] Zakani B., Entezami S., Grecov D., Salem H., Sedaghat A. (2022). Effect of Ultrasonication on Lubrication Performance of Cellulose Nano-Crystalline (CNC) Suspensions as Green Lubricants. Carbohydr. Polym..

[34] Browne C., Raghuwanshi V.S., Lin M., Garnier G., Batchelor W. (2022). Characterisation of Cellulose Nanocrystals by Rheology and Small Angle X-Ray Scattering (SAXS). Colloids Surf. A Physicochem. Eng. Asp..

[35] Chen Y.W., Lee H.V., Juan J.C., Phang S.-M. (2016). Production of New Cellulose Nanomaterial from Red Algae Marine Biomass Gelidium Elegans. Carbohydr. Polym..

[36] El Achaby M., El Miri N., Hannache H., Gmouh S., Aboulkas A. (2018). Production of Cellulose Nanocrystals from Vine Shoots and Their Use for the Development of Nanocomposite Materials. Int. J. Biol. Macromol..

[37] Jonoobi M., Oladi R., Davoudpour Y., Oksman K., Dufresne A., Hamzeh Y., Davoodi R. (2015). Different Preparation Methods and Properties of Nanostructured Cellulose from Various Natural Resources and Residues: A Review. Cellulose.

[38] Hedjazi S., Razavi S.H. (2018). A Comparison of Canthaxanthine Pickering Emulsions, Stabilized with Cellulose Nanocrystals of Different Origins. Int. J. Biol. Macromol..

[39] Kumar A., Negi Y.S., Choudhary V., Bhardwaj N.K. (2014). Characterization of Cellulose Nanocrystals Produced by Acid-Hydrolysis from Sugarcane Bagasse as Agro-Waste. J. Mater. Phys. Chem..

[40] Ilyas R.A., Sapuan S.M., Ishak M.R. (2018). Isolation and Characterization of Nanocrystalline Cellulose from Sugar Palm Fibres (Arenga Pinnata). Carbohydr. Polym..

[41] Beck-Candanedo S., Roman M., Gray D.G. (2005). Effect of Reaction Conditions on the Properties and Behavior of Wood Cellulose Nanocrystal Suspensions. Biomacromolecules.

[42] Yu L., Lin J., Tian F., Li X., Bian F., Wang J. (2014). Cellulose Nanofibrils Generated from Jute Fibers with Tunable Polymorphs and Crystallinity. J. Mater. Chem. A.

[43] Gong J., Li J., Xu J., Xiang Z., Mo L. (2017). Research on Cellulose Nanocrystals Produced from Cellulose Sources with Various Polymorphs. RSC Adv..

[44] Chen Q., Liu P., Nan F., Zhou L., Zhang J. (2014). Tuning the Iridescence of Chiral Nematic Cellulose Nanocrystal Films with a Vacuum-Assisted Self-Assembly Technique. Biomacromolecules.

[45] Csiszar E., Kalic P., Kobol A., Ferreira E.d.P. (2016). The Effect of Low Frequency Ultrasound on the Production and Properties of Nanocrystalline Cellulose Suspensions and Films. Ultrason. Sonochem..

[46] Bao Y., Liu K., Zheng Q., Yao L., Xu Y. (2022). A Review of Preparation and Tribological Applications of Pickering Emulsion. J. Tribol..

[47] Girard M., Bertrand F., Tavares J.R., Heuzey M.-C. (2021). Rheological Insights on the Evolution of Sonicated Cellulose Nanocrystal Dispersions. Ultrason. Sonochem..

[48] Beck S., Bouchard J. (2016). Ionic Strength Control of Sulfated Cellulose Nanocrystal Suspension Viscosity. TAPPI J..

[49] Shafiei-Sabet S., Hamad W.Y., Hatzikiriakos S.G. (2012). Rheology of Nanocrystalline Cellulose Aqueous Suspensions. Langmuir.

[50] Kádár R., Spirk S., Nypelö T. (2021). Cellulose Nanocrystal Liquid Crystal Phases: Progress and Challenges in Characterization Using Rheology Coupled to Optics, Scattering, and Spectroscopy. ACS Nano.

[51] Schutz C., Agthe M., Fall A.B., Gordeyeva K., Guccini V., Salajková M., Plivelic T.S., Lagerwall J.P., Salazar-Alvarez G., Bergstrom L. (2015). Rod Packing in Chiral Nematic Cellulose Nanocrystal Dispersions Studied by Small-Angle X-Ray Scattering and Laser Diffraction. Langmuir.

[52] Miller D.S., Carlton R.J., Mushenheim P.C., Abbott N.L. (2013). Introduction to Optical Methods for Characterizing Liquid Crystals at Interfaces. Langmuir.

[53] Casado U., Mucci V.L., Aranguren M.I. (2021). Cellulose Nanocrystals Suspensions: Liquid Crystal Anisotropy, Rheology and Films Iridescence. Carbohydr. Polym..

[54] Honorato-Rios C., Kuhnhold A., Bruckner J.R., Dannert R., Schilling T., Lagerwall J.P.F. (2016). Equilibrium Liquid Crystal Phase Diagrams and Detection of Kinetic Arrest in Cellulose Nanocrystal Suspensions. Front. Mater..

[55] Wu J., Ma G.-H. (2016). Recent Studies of Pickering Emulsions: Particles Make the Difference. Small.

[56] Binks B.P., Lumsdon S.O. (2000). Influence of Particle Wettability on the Type and Stability of Surfactant-Free Emulsions. Langmuir.

[57] Sarkar A., Dickinson E. (2020). Sustainable Food-Grade Pickering Emulsions Stabilized by Plant-Based Particles. Curr. Opin. Colloid Interface Sci..

[58] Jakubek Z.J., Chen M., Couillard M., Leng T., Liu L., Zou S., Baxa U., Clogston J.D., Hamad W.Y., Johnston L.J. (2018). Characterization Challenges for a Cellulose Nanocrystal Reference Material: Dispersion and Particle Size Distributions. J. Nanoparticle Res..

[59] Niroula A., Gamot T.D., Ooi C.W., Dhital S. (2021). Biomolecule-Based Pickering Food Emulsions: Intrinsic Components of Food Matrix, Recent Trends and Prospects. Food Hydrocoll..

[60] Yang C., Li J., Zhang Y., Wu C., Li D. (2023). A Pesticide Sustained-Release Microcapsule from Cellulose Nanocrystal Stabilized Pickering Emulsion Template. J. Appl. Polym. Sci..

[61] Ng S.-W., Chong W.-T., Soo Y.-T., Tang T.-K., Karim N.A.A., Phuah E.-T., Lee Y.-Y. (2022). Pickering Emulsion Stabilized by Palm-Pressed Fiber Cellulose Nanocrystal Extracted by Acid Hydrolysis-Assisted High Pressure Homogenization. PLoS ONE.

[62] Ataeian P., Aroyan L., Parwez W., Tam K.C. (2022). Emulsions Undergoing Phase Transition: Effect of Emulsifier Type and Concentration. J. Colloid Interface Sci..

[63] Kinra S., Pal R. (2023). Rheology of Pickering Emulsions Stabilized and Thickened by Cellulose Nanocrystals over Broad Ranges of Oil and Nanocrystal Concentrations. Colloids Interfaces.

[64] Miao C., Mirvakili M.-N., Hamad W.Y. (2022). A Rheological Investigation of Oil-in-Water Pickering Emulsions Stabilized by Cellulose Nanocrystals. J. Colloid Interface Sci..

[65] Miao C., Tayebi M., Hamad W.Y. (2018). Investigation of the Formation Mechanisms in High Internal Phase Pickering Emulsions Stabilized by Cellulose Nanocrystals. Philos. Trans. R. Soc. A Math. Phys. Eng. Sci..

[66] Zhang Z., Cheng M., San Gabriel M., Neto Â.A.T., da Silva Bernardes J., Berry R., Tam K.C. (2019). Polymeric Hollow Microcapsules (PHM) via Cellulose Nanocrystal Stabilized Pickering Emulsion Polymerization. J. Colloid Interface Sci..

[67] Martins D., Estevinho B., Rocha F., Dourado F., Gama M. (2020). A Dry and Fully Dispersible Bacterial Cellulose Formulation as a Stabilizer for Oil-in-Water Emulsions. Carbohydr. Polym..

[68] Chu G., Vasilyev G., Vilensky R., Boaz M., Zhang R., Martin P., Dahan N., Deng S., Zussman E. (2018). Controlled Assembly of Nanocellulose-Stabilized Emulsions with Periodic Liquid Crystal-in-Liquid Crystal Organization. Langmuir.

[69] Wan W., Zhao Z., Hughes T.C., Qian B., Peng S., Hao X., Qiu J. (2015). Graphene Oxide Liquid Crystal Pickering Emulsions and Their Assemblies. Carbon.

